# A Review of Progress on Targeting LDL Receptor-Dependent and -Independent Pathways for the Treatment of Hypercholesterolemia, a Major Risk Factor of ASCVD

**DOI:** 10.3390/cells12121648

**Published:** 2023-06-16

**Authors:** Rai Ajit K. Srivastava

**Affiliations:** 1 Integrated Pharma Solutions LLC, Boston, MA 02101-02117, USA; ajitsriva@gmail.com; Tel.: +1-215-801-5129; 2 College of Professional Studies, Northeastern University, Boston, MA 02101-02117, USA

**Keywords:** hypercholesterolemia, LDL receptor, PCSK9, ANGPTL3, CRISPR-Cas9, atherosclerosis, ASCVD, genome editing

## Abstract

Since the discovery of the LDL receptor in 1973 by Brown and Goldstein as a causative protein in hypercholesterolemia, tremendous amounts of effort have gone into finding ways to manage high LDL cholesterol in familial hypercholesterolemic (HoFH and HeFH) individuals with loss-of-function mutations in the LDL receptor (LDLR) gene. Statins proved to be the first blockbuster drug, helping both HoFH and HeFH individuals by inhibiting the cholesterol synthesis pathway rate-limiting enzyme HMG-CoA reductase and inducing the LDL receptor. However, statins could not achieve the therapeutic goal of LDL. Other therapies targeting LDLR include PCSK9, which lowers LDLR by promoting LDLR degradation. Inducible degrader of LDLR (IDOL) also controls the LDLR protein, but an IDOL-based therapy is yet to be developed. Among the LDLR-independent pathways, such as angiopoietin-like 3 (ANGPTL3), apolipoprotein (apo) B, apoC-III and CETP, only ANGPTL3 offers the advantage of treating both HoFH and HeFH patients and showing relatively better preclinical and clinical efficacy in animal models and hypercholesterolemic individuals, respectively. While loss-of-LDLR-function mutations have been known for decades, gain-of-LDLR-function mutations have recently been identified in some individuals. The new information on gain of LDLR function, together with CRISPR-Cas9 genome/base editing technology to target LDLR and ANGPTL3, offers promise to HoFH and HeFH individuals who are at a higher risk of developing atherosclerotic cardiovascular disease (ASCVD).

## 1. Introduction

**Figure 1:**
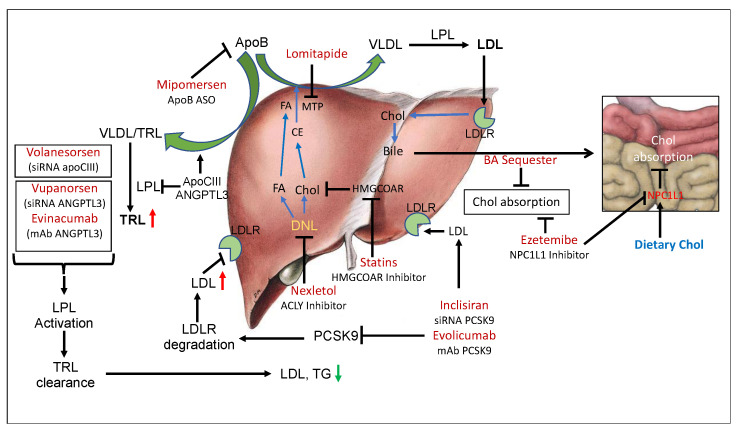
LDLR-dependent and -independent pathways to manage circulating LDL levels. De novo lipogenesis (DNL) provides fatty acids and cholesterol for the assembly of apoB-containing very low-density lipoproteins (VLDL), where apoB serves as the main protein and microsomal triglyceride transfer protein (MTP) facilitates the assembly of VLDL. By inhibiting DNL by Nexletol (ACLY inhibitor), it is possible to reduce the synthesis and secretion of VLDL. Inhibition of MTP by lomitapide and silencing of apoB production by mipomersen also restricts VLDL secretion and leads to reduced levels of LDL. Dietary cholesterol absorption by bile acid (BA) sequesters and ezetimibe, an NPC1L1 inhibitor, also reduces circulating LDL. Other pathways to reduce LDL are to inhibit the degradation of LDLR by PCSK9 and enhance clearance of LDL. Inclisiran (PCSK9 ASO) and evolocumab (PCSK9 mAb) enhance LDL clearance. LDL and triglyceride-rich lipoproteins can also be removed by silencing apoCIII and ANGPTL3 by using volanesorsen (apoCIII ASO), vupanorsen (ANGPTL3 ASO), and evinacumab (ANGPTL3 mAb). DNL, de novo lipogenesis; BA, bile acid; TRL, triglyceride-rich lipoproteins; LPL, lipoprotein lipase; FA, fatty acid; CE, cholesterol ester; MTP, microsomal triglyceride transfer protein. Green arrows indicate heepatic synthesis and secretion of lipoproteins. Red arrows indicate CVD risk.

Familial hypercholesterolemia (FH) is one of the prevalent and widely studied monogenic metabolic disorders caused by mutations in the low-density lipoprotein receptor (LDLR) gene [1,2,3], with a frequency estimated at 1:170,000 to 1:300,000 [4]. Other gene mutations combined with LDLR mutations cause severe hypercholesterolemia [5]. While hypercholesterolemia has been reported worldwide, it is one of the most common genetic diseases in the Polish population, where the estimated incidence of heterozygous familial hypercholesterolemia (HeFH) is around 1:250. Recent results showed that as many as 1.6% (1 in 63) of patients with very high cardiovascular disease (CVD) risk have HeFH [6], showing a direct correlation between LDLR and LDL cholesterol leading to CVD. Both homozygous hypercholesterolemia (HoFH) and HeFH are characterized by a high level of atherogenic LDL cholesterol, leading to premature atherosclerotic cardiovascular disease (ASCVD) [7]. Both heterozygous and homozygous FH patients develop premature coronary heart disease (CHD). It is possible to reduce LDL cholesterol (LDL) by one of two pathways: (a) production of LDL; and (b) clearance of LDL from circulation. Several players influence LDL production; some of them are (a) cholesterol synthesis enzymes such as β-Hydroxy β-methylglutaryl-CoA reductase (HMG-COAR); (b) ATP citrate lyase (ACLY), which inhibits both cholesterol and fatty acid synthesis [8,9]; (c) apolipoprotein B (apoB), the main protein component of LDL [10]; and (d) cholesterol ester transfer protein (CETP) [11,12]. The players responsible for the clearance of VLDL and LDL particles from circulation include the LDL receptor, the main pathway of LDL clearance from circulation [13,14], and the VLDL receptor [15]. Inhibition of proprotein convertase subtilisin/kexin type 9 (PCSK-9) [16,17], which degrades LDLR protein, and inducible degrader of LDLR (IDOL) [18] both inhibit LDL clearance. Inactivation of apolipoprotein CIII (apoC-III) [19,20] and the angiopoietin-like 3 (ANGPTL3) protein [21,22] lowers LDL and triglycerides (TG) by enhancing the clearance of TG-rich lipoproteins (TLRs). In addition to the above pathways, another mechanism, independent of LDL production and LDL clearance, also influences circulating LDL through limiting the absorption of intestinal cholesterol [23,24], which is mediated via the Niemann–Pick C1-Like1 (NPC1L1) protein expressed predominantly in the gut [25] (Figure 1, Table 1).

The marketed agents known to lower LDL in hypercholesterolemic individuals include statins [26,27,28], ezetimibe [29,30], bile acid sequestrants [31,32,33], niacin [34,35,36,37], lomitapide [38], mipomersen [39,40], low-density lipoprotein (LDL) apheresis [41] and Nexletol [42,43,44] (Figure 1, Table 1). Early treatment by statins has been shown to result in a substantial reduction in cardiovascular events and death in patients with familial hypercholesterolemia [26,45]. Novel therapies not primarily dependent on the functioning of LDLR include lomitapide [38,46] and mipomersen [39,40,46,47], which decrease hepatic apolipoprotein B secretion, and evinacumab [48,49,50,51], directed at the angiopoietin like-3 protein (ANGPLT-3), which enhances clearance of highly atherogenic TLRs [52,53,54] and lowers the risk of ASCVD [55,56]. Similarly, antisense therapy with volanesorsen targeting apoC-III [20,57,58,59] enhances lipoprotein lipase (LPL) activity such as ANGPTL3 [60] and accelerates the clearance of TLRs. ACLY inhibitor, which inhibits both cholesterol and fatty acids synthesis [8,9] and is also known to induce hepatic LDLR [61], is a recent addition to cholesterol-lowering therapy [42,44,62,63]. As far as the clearance of LDL particles is concerned, the LDL receptor is the predominant player that determines LDL concentrations in the circulation and LDL levels that correlate to CVD risk [64]. Evidence from genetic, epidemiological, and clinical studies shows LDL as a causative agent for ASCVD [7]; however, certain pathophysiological conditions observed in diabetic patients, LDLR activity, and LDL levels do not solely determine cardiovascular risk since other factors contribute to endothelial dysfunction and increased risk of ASCVD [65]. Advanced glycated end products (AGEs) in diabetes cause oxidative stress [66], leading to elevated levels of oxidized LDL (oxLDL), which is proatherogenic and also activates inflammatory responses [67]. So, in certain pathological conditions, instead of LDL, oxLDL correlates better with ASCVD [68]. High-density lipoprotein (HDL) possesses anti-inflammatory properties and is cardioprotective; however, in diabetic individuals, HDL undergoes oxidative modification and becomes proinflammatory and dysfunctional [69]. LDL particle size also determines CVD risk [70], and in patients with low LDL (<100 mg/dL), lipoproteins(a) (Lp(a)) is a better predictor of CVD risk than LDL [71]. LDL receptor activity has been reported to influence blood LDL levels under the following situations: (a) HMG-CoA reductase inhibitors increase LDLR activity both in animal models and in humans, and reduce lesion formation; (b) PCSK9 inhibition increases LDLR activity in animal models and in humans and reduces arterial lesion formation in animal models; (c) IDOL suppression induces LDLR activity in HepG2 cells and animal models; and (d) ATP citrate lyase (ACLY) inhibition increases LDLR activity in animal models and humans and reduces lipid deposition in the arterial wall. Both antisense oligos (ASOs) [72,73] and monoclonal antibodies mAbs [74,75,76,77] have been developed for PCSK9-based therapy. Similarly, both ASO-based [59] and mAB-based [51] therapies targeting ANGPTL3 have been developed.

**Table 1:** Approved lipid-lowering therapies targeting LDLR-dependent and -independent pathways that resulted in a reduction in CVD risk, which corroborated the loss- or gain-of-function mutations in humans (discussed in the text).

Drug Name	Drug Class	Primary Endpoint	Mechanism	Refs.
Statins	LDL lowering	LDL	HMG-CoA Inhibitor	[27,28]
Cholestyramine	Bile acid sequestrants	LDL	Bile acid binding	[32,33]
Apheresis		LDL	Apheresis	[78]
Niacin	LDL lowering	TG, LDL HDL	DGAT2 Inhibitor	[34,35]
Ezetimibe	LDL Lowering	LDL	NPC1L1 inhibitor	[29,30]
Lomitapide	LDL lowering	VLDL, LDL	MTP Inhibitor	[46]
Nexletol	LDL lowering	LDL	ACLY Inhibitor	[42,44]
Mipomersen	ASO	LDL	ApoB silencing	[46,47]
Volanesorsen	siRNA	TG	ApoCIII silencing	[58,59]
ARO-ApoCIII	siRNA	TG	ApoCIII silencing	[59]
Vupanorsen	siRNA	LDL, TG	ANGPTL3 silencing	[21]
IONIS-ANGPTL3	ASO	LDL, TG	ANGPTL3 silencing	[59]
ARO-ANG3	siRNA	LDL, TG	ANGPTL3 silencing	[59]
Evinacumab	mAb	LDL, TG	ANGPTL3 silencing	[51]
Inclisiran	siRNA	LDL	PCSK9 silencing	[72,73]
Evolocumab	mAb	LDL	PCSK9 silencing	[74,75]
Alirocumab	mAb	LDL	PCSK9 silencing	[76,77]

## 2. LDLR Pathway Controlling LDL Level

**Figure 2:**
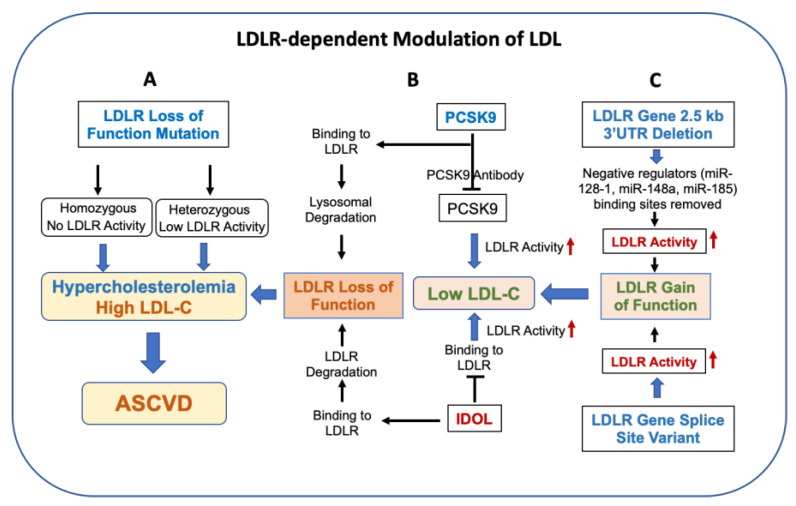
LDLR modulates circulating levels of LDL and lowers the risk of ASCVD. Panel (**A**): LDLR loss-of-activity mutation in humans is associated with elevated LDL, resulting in familial hypercholesterolemia (HoFH and HeFH) and an increase in the risk of ASCVD. Panel (**B**): PCSK9 and IDOL also regulate LDLR activity via degradation of the LDLR protein. Inhibition of PCSK9 and IDOL by mAb increases LDL activity and decreases circulating LDL. Panel (**C**): Gain of LDLR activity as a result of splice site variants and deletion of 3′ UTR lowers circulating LDL. The 2.5 kb LDLR 3′UTR sequences contain binding sites of miRNAs [89,90,91,92,93] known to repress LDLR expression.

Low levels of LDLR activity and high circulating LDL cholesterol were first demonstrated in vitro in the fibroblasts isolated from FH patients [79]. Lowering of LDL by statins [80] and an association between LDLR activity and circulating LDL in HeFH were subsequently reported [81]. Circulating cholesterol reduction by pravastatin was associated with the induction of LDLR activity in isolated skin fibroblasts [82]. Statins lower LDL in animal models [83] as well as in humans [45] by inhibiting HMG-COA reductase, a key enzyme in the cellular cholesterol synthesis pathway. This causes the depletion of liver cholesterol, leading to the induction of LDLR gene expression [84]. Thus, statin therapy, on one hand, reduces cholesterol production, and on the other hand induces LDL receptor expression, resulting in enhanced clearance of atherogenic LDL lipoproteins. Several classes of drug products have been used since the launch of statins to treat hypercholesterolemia in humans and many of these approaches of cholesterol lowering involve inducing LDL receptor activity (Table 1, Figure 2). One of the players studied in detail is proprotein convertase subtilisin/kexin type 9 (PCSK9) [85]. PCSK9 is a serine protease belonging to the proprotein convertase family, mainly produced by the liver, and essential for metabolism of LDL particles by inhibiting LDLR recirculation to the cell, causing the elevation of LDL-C levels by inducing LDL receptor protein degradation via clathrin-mediated endocytosis and routing to lysosomes via a mechanism that does not require ubiquitination and is distinct from the autophagy and proteasomal degradation pathways [86]. Thus, loss of function of PCSK-9 is associated with low LDL [87,88]. Gain of LDLR activity as a result of splice site variants and deletion of 3′ UTR lowers circulating LDL. The 2.5 kb LDLR 3′UTR sequences contain binding sites of miRNAs [89,90,91,92,93] known to repress LDLR expression. A more detailed discussion on LDL-dependent pathways is provided in Section 3 of this review. 

### 2.1. LDLR-Independent Pathway Affecting LDL Level

**Figure 3:**
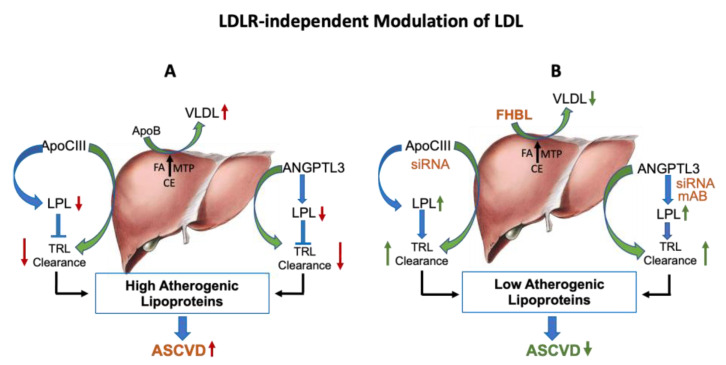
LDLR-independent pathways regulate circulating LDL. (**A**): Liver synthesizes and secretes VLDL lipoproteins in the circulation. Intrahepatic assembly of VLDL requires apoB, MTP and triglycerides. ApoC-III is a component of VLDL and chylomicron remnants, and also an inhibitor of lipoprotein lipase. Another protein, ANGPTL3, also facilitates secretion of TRL from the liver and impairs clearance of TRL. Thus, apoB, apoC-III and ANGPTL3 increase the pro-atherogenic lipoproteins. (**B**): Hepatic VLDL secretion can be lowered by the reduced production of apoB. In FHBL individuals, the nonsense mutation in the apoB gene results in low levels of apoB, resulting in less secretion of VLDL. ApoC-III and ANGPTL3 antisense therapies both influence hepatic secretion of TRL and increase LPL activity, leading to reductions in apoB-containing lipoproteins and TG. Green color arrows indicate production of lipoproteins and blue color arrows indicate clearance of lipoproteins.

Among LDLR-independent pathways, suppressing apoB [94] production and inhibiting the function of apoC-III [95] and angiopoietin like-3 protein (ANGPTL-3) [52,55] have been studied in greater detail [96] (Figure 3). ApoB is a large 550 kDa protein and is essential for the assembly and secretion of VLDL by the liver [10,97]. VLDL undergoes lipolysis to form LDL [98,99]. ApoB also serves as a ligand for LDLR-mediated cellular uptake by the peripheral tissues and clearance from circulation via the liver [69]. While suppression of apoB synthesis in the liver leads to reductions in the apoB-containing atherogenic lipoproteins [47], it may cause fatty liver as a result of accumulation of fat in the liver, as seen in familial hypobetalipoproteinemia (FHBL) individuals with reduced levels of apoB [100,101], partly as a result of enhanced intracellular degradation of apoB [102]. FHBL individuals are also reported to have intestinal lipid accumulation and postprandial lipemia [103], which in some cases is associated with the risk of diabetes [104]. The hypolipidemic actions of both apoC-III and ANGPTL3 inhibitors are associated with lowering of atherogenic TLRs [59,105] via activation of lipoprotein lipase (LPL) [106,107,108,109]. LPL is an important player in the metabolism of TLRs [47], resulting in cardio-protection [110]. Human apoC-III is a small apoprotein with a molecular weight of 10 kDa, predominantly synthesized in the liver and intestine and primarily associated with chylomicrons, VLDL and HDL [111]. ApoC-III is an inhibitor of LPL that hydrolyzes TG-rich VLDL and impairs its clearance [108]. Thus, inactivation of apoC-III should prove beneficial to individuals with hyperlipidemia. Indeed, mice lacking apoC-III are shown to have increased LPL activity and enhanced lipolysis of TRLs and show lack of postprandial lipemia [112]. In rodents and non-human primates, apoC-III ASOs lowered plasma apoC-III and TG [95]. Silencing apoC-III activity via antisense therapy lowered TRLs in diabetic [113] and non-diabetic [114] individuals as a result of enhanced LPL activity [20]. Despite increased lipolysis of TRLs by LPL and reduced TG in apoC-III-deficient mice [112], no difference in atherosclerosis burden was noticed in the apoC-III-deficient mice compared to the control on LDLR-deficient background [115], although humans with apoC-III loss-of-function mutations showed apparent cardio-protection in carriers compared to non-carriers, based on subclinical atherosclerosis findings measured by calcium score [116]. Rare variants of apoC-III loss-of-function mutations in humans have also been reported to provide cardio-protection [117,118]. Inhibition of apoC-III accelerates its clearance of TRLs from circulation and reduces the risk of ASCVD. Lowering or loss of function of apoCIII results in the reduced secretion of chylomicron remnants and activation of LPL, leading to enhanced clearance of atherogenic lipoproteins [119]. Thus, apoCIII increases production of chylomicron remnants and also impedes clearance of TG-rich apoB lipoproteins. However, apoC-III antisense therapy is associated with increased risk of thrombocytopenia [120]. Newer apoC-III antagonists such as ASO olezarsen (formerly AKCEA-APOCIII-LRx) and short interfering RNA (siRNA) ARO-APOC3 appear to show efficacy with less risk of thrombocytopenia [120].

ANGPTL3 is a 460-amino-acid secretory glycoprotein expressed in the liver and implicated in the metabolism of lipids through modulation of LPL. ANGPTL3 inhibits lipoprotein lipase (LPL) [60] and endothelial lipase (EL) [121] in tissues, leading to formation of TRLs [122,123,124,125]. Thus, inactivation of ANGPTL3 leads to LPL activation and enhanced clearance of TRLs from the circulation [60]. ANGPTL3 inhibition also reduces hepatic VLDL secretion [126], resulting in the lowering of blood LDL and TG. Indeed, loss-of-function mutations affecting the gene encoding ANGPTL3 are linked with lower total cholesterol, LDL-C and TG levels and provide cardio-protection [22,55,127]. Consistent with the human data, ANGPTL3-null mice showed lower LDL and TG as a result of enhanced LPL activity and accelerated clearance of TRLs [106]. ANGPTL3 antibodies have been found effective in reducing LDL in humans lacking LDLR (HoFH), where LDLR-dependent therapies have been ineffective [128]. Thus, ANGPTL3 offers a unique opportunity to lower atherogenic lipoproteins in patients not able to benefit from LDLR-dependent therapies. ApoC-III and ANGPTL3 are discussed further in Section 4 of this review.

### 2.2. LDLR Activity and Circulating LDL

In vivo proof-of-concept as well as pharmacology studies of therapeutic agents (new chemical entities (NCEs)/new biological entities (NBEs)), are first evaluated in relevant animal models since testing agents may show species [129,130,131] and strain [132] specificity, which can be examined using mammalian cells derived from animals. These studies are designed to determine various parameters for pharmacological effects, which assist in the selection of appropriate animal species for further in vivo pharmacology and toxicology studies. The combined results from in vitro and in vivo studies assist in the extrapolation of the findings to humans. In vivo studies to assess pharmacological activity, including defining mechanism(s) of action, are often used to support the rationale of the proposed use of the drug product in clinical studies. Several animal models have been described to assess atherosclerosis in hypercholesterolemic conditions [133,134]. Some examples of animal models that have been used to determine the association between LDL receptor activity and circulating LDL are described in pharmacology studies below. 

### 2.3. LDLR Activity Determines Circulating LDL

In mice, a clear association between LDLR activity and circulating LDL levels was demonstrated by either overexpressing the LDL receptor or making LDL-receptor-deficient mice (Figure 2). Hofmann et al. [135] generated a transgenic mouse model expressing the human LDL receptor gene. These transgenic mice cleared intravenously injected ^125^I-labeled LDL from blood eight to ten times more rapidly than normal mice. The plasma concentrations of apoproteins B-100 and E, the two ligands for the LDL receptor, declined by more than 90 percent, but the concentration of another apoprotein, apoA-I, was unaffected. These studies demonstrated that overexpression of the LDL receptor can dramatically lower the concentration of LDL in vivo. To further confirm that, indeed, an association exists between LDL receptor activity and circulating LDL concentration, the same group of researchers [13] generated a mouse model lacking the LDL receptor by homologous recombination. Total plasma cholesterol levels were two-fold higher in homozygous mice than those of wild-type littermates, owing to a seven- to nine-fold increase in atherogenic lipoproteins (IDL and LDL). No significant change in HDL was observed. Despite the mouse lipoprotein profile differing from that of humans, the LDLR-mediated regulation of circulating LDL appears to be similar between mice and humans in terms of LDLR-mediated clearance of LDL lipoproteins. The half-lives of intravenously administered ^125^I-VLDL and ^125^I-LDL were prolonged by 30-fold and 2.5-fold, respectively. Intravenous injection of a recombinant replication-defective adenovirus encoding the human LDL receptor restored expression of the LDL receptor protein in the liver and increased the clearance of ^125^I-VLDL, further confirming the inverse association of LDL receptor activity and LDL level and the role of the LDL receptor in reversing the hypercholesterolemic effects of LDL receptor deficiency. An LDLR deficient mouse model has been extensively employed to ask important biologic questions, including the mechanism of atherosclerosis progression by nuclear hormone receptors [136,137,138], HDL receptors [139] and players in the cholesterol efflux pathway [140].

#### 2.3.1. LDLR Activity and LDL Association in Mice and Rats

Both LDLR [13] as well as apoE knockout mice [141,142] develop hypercholesterolemia and atherosclerosis, the latter showing more severe symptoms than the former. Using these mice, mechanisms of atherogenesis have been studied extensively [143,144,145]. WT rats and mice develop very sparse atherosclerotic lesions, even on high-fat and high-cholesterol diets. Sithu et al. [146] developed an LDLR KO model in Sprague-Dawley rats and studied lipoprotein metabolism and atherosclerotic lesion formation. Lack of LDLR protein in rats caused a significant increase in plasma total cholesterol and triglycerides; the rats gained more weight and were more glucose intolerant than WT rats on normal rat chow diet. Feeding a Western diet resulted in increased obesity and age-dependent increases in glucose intolerance, as well as significant atherosclerotic lesions in the aortic arch as well as throughout the abdominal aorta in the LDLR KO rats. These findings demonstrated a tight association between LDLR and LDL.

#### 2.3.2. Regulation of LDL in LDLR-Deficient Hamsters

The golden Syrian (GS) hamster has been used with increasing frequency to study lipoprotein metabolism [147,148] and atherosclerosis [149,150] and to evaluate hypolipidemic agents [9,150] because of the hamster’s lipid metabolism being comparable to that of humans. The VLDL particles synthesized and secreted by hamster liver are similar to human liver with apoB-100 [97,151], but different than rats and mice that secrete both apoB-100 and B-48 [152]. As in humans, hamsters also show CETP activity in plasma [151]. These characteristics of hamsters make them a suitable animal model for evaluating lipid-modulating agents in preclinical studies. Hamsters also respond to high-cholesterol and high-fat diets similarly to humans [153] and develop more atherosclerosis compared to rats and mice [154]. These features of hamsters make them very useful to test hypolipidemic agents. Because of the similarity of hamster and human lipoprotein metabolism, an LDL-receptor-deficient hamster model was developed by Guo et al. [155] using CRISPR-Cas9 technology. Homozygous LDLR KO hamsters on a chow diet developed hypercholesterolemia with LDL as the dominant lipoprotein and spontaneous atherosclerosis. On a high-cholesterol, high-fat (HCHF) diet, these animals exhibited severe hyperlipidemia and atherosclerotic lesions in the aorta and coronary arteries. Moreover, the heterozygous LDLR KO hamsters on a short-term HCHF diet also had apparent hypercholesterolemia, which could be effectively ameliorated with several lipid-lowering drugs. Importantly, heterozygotes fed HCHF diets for 3-months developed accelerated lesions in their aortas and coronary arteries [156], whereas only mild aortic lesions were observed in WT hamsters. Thus, unlike other rodent animals, the levels of plasma cholesterol in hamsters can be significantly modulated by the intervention of dietary cholesterol, levels which were closely associated with severity of atherosclerosis in LDLR^+/−^ hamsters. Further validation of the heterozygous hamster (LDLR^+/−^) model for testing therapeutic agents was shown in a study where hyperlipidemic heterozygous hamsters were treated with the PCSK9 monoclonal antibody [157]. In this study, either evolocumab or ezetimibe treatment prevented high-fat-diet-induced hyperlipidemia and atherosclerotic plaque formation. Thus, the LDL receptor gene copy number influences plasma LDL concentrations and suggests an association between the LDL receptor and circulating LDL. 

#### 2.3.3. LDLR-Deficient Rabbits as a Model of HoFH

Familial hypercholesterolemia (FH) is caused primarily by loss-of-function mutations in the LDLR gene [79,80], leading to elevated concentrations of LDL in heterozygous and, most notably, homozygous patients [14,55,158]. FH is also associated with significantly reduced concentrations of both high-density lipoprotein cholesterol (HDL-C) and its principal protein component, apolipoprotein (apo) A-I [159], as a result of increased apoA-I catabolism and decreased apoA-I synthesis [160]. A rabbit model for FH, the Watanabe heritable hyperlipidemic (WHHL) rabbit, was discovered and reported in 1980 [161,162]. These rabbits have an LDLR gene that encodes a four-amino-acid deletion in the cysteine-rich ligand-binding domain of the protein that severely disrupts LDLR function [163]. Homozygous WHHL rabbits are markedly hypercholesterolemic from birth and suffer from tendon xanthomas and atherosclerosis, both of which exhibit remarkable pathological resemblance to those observed in human HoFH. Also similar to the human condition [159], plasma HDL-C and apoA-I levels are abnormally low in these animals [163]. Overexpression of human lecithin: cholesterol acyltransferase (hLCAT), a pivotal enzyme involved in HDL metabolism, in LDLR defective rabbits increased HDL-C and apoA-I levels [160]. Similar to humans [82], treatment with statins induced LDL receptor expression in WHHL heterozygotes [164], suggesting the WHHL rabbit as a useful LDLR-deficient model that mimics human HoFH.

#### 2.3.4. LDLR Loss-of-Function Studies in Pig

Yucatan miniature pigs are well established as translational research models because of similarities to humans in physiology, anatomy, genetics and size of the atherosclerotic plaques. The pig LDL receptor gene was inactivated to drive hypercholesterolemia and atherosclerosis by two groups of researchers using separate technologies [165,166]. These mini pigs with an ablated LDLR gene showed diet-induced exacerbation of FH phenotypes. Hypercholesterolemic heterozygotes (LDLR^+/−^) showed similar characteristics as human heterozygotes (LDLR^+/−^) and a similar response when treated with statins (atorvastatin, 3 mg/kg/day). Homozygous (LDLR^−/−^) pigs also showed human-like advanced plaques and responded to statin (pitavastatin) treatment [167]. In the homozygous (LDLR^−/−^) and heterozygotes (LDLR^+/−^) Yucatan miniature pigs, another class of drug candidate, bempedoic acid, which inhibits ATP citrate lyase, showed a reduction in LDL and lesion formation [168]. The ablation of LDLR caused elevation of LDL in homozygous and heterozygous miniature pigs, suggesting an association between LDL receptor function and LDL concentration.

## 3. LDLR Degradation Pathway Regulates LDL

### 3.1. PCSK9 Regulation of LDLR and LDL

Since circulating LDL levels are associated with LDLR activity, any agents that disrupt the function of LDLR can result in the elevation of LDL (Figure 1). Therefore, pathways that degrade the LDL receptor protein can lead to hypercholesterolemia. This class of therapeutic targets includes PCSK9, a member of the proteinase K subfamily of subtilases that reduces the number of LDL receptors (LDLRs) in liver through endocytosis [17]. Further evidence of LDL lowering by LDLR degradation pathways was demonstrated in a combination study of the PCSK9 monoclonal antibody with statins that showed increases in the LDL receptor, more than either treatment alone, in HepG2 cells [169], confirming that PCSK9 and statins influence LDLR by an independent mechanism. PCSK9 mAb1 lowered serum cholesterol by 50% at a 10 mg/kg dose in WT C57Bl mice, which was associated with two-fold increases in LDLR protein, and in cynomolgus monkey, a single injection of mAb1 reduced serum LDL-C by 80%, a significant decrease that was maintained for 10 days [169]. Notably, LDL-C and free PCSK9 levels seemed to return to pre-dose levels at a similar rate. Thus, PCSK9-mediated degradation of LDLR and serum LDL in cynomolgus monkey showed a tight correlation. Indeed, a mouse model lacking PCSK9 showed protection against developing aortic lesions when fed a Western diet as a result of lower atherogenic lipoproteins compared to WT littermates, and mice overexpressing PCSK9 showed a three-fold greater increase in aortic lesions compared to WT littermates [88]. In a separate carotid ligation mouse model [170], PCSK9 overexpression using AAV-PCSK9 led to elevated serum PCSK9, hypercholesterolemia and rapid atherosclerosis development within 3 weeks when compared to the control. As expected, loss-of-function mutation of the *PCSK9* gene in humans showed low levels of LDL [87] and protection against coronary artery disease [171,172]. On the contrary, a gain-of-function mutation in the PCSK9 gene (Arg499 → His) leads to familial hypercholesterolemia as a result of LDL receptor degradation [173]. More complex compound mutations in LDLR and PCSK9 genes have also been reported. In one study [172], the genetic analysis showed a pathogenic heterozygous mutation in LDLR [exon 6:c.902A>G:p (Asp301Gly)], as well as a loss-of-function heterozygous variant in PCSK9 [exon1:c.137 G>T:p.(Arg46Leu)]. In this subject, loss-of-function mutation in the LDLR gene tended to cause hypercholesterolemia, but the loss-of-function mutation in PCSK9 offered protected from hypercholesterolemia because of the unaffected LDLR allele that produced the LDLR protein. Thus, all evidence suggests a tight association between LDLR function and circulating LDL. Several investigators have provided clinical evidence in support of such an association in studies designed to inhibit PCSK9 in hypercholesterolemic patients by monoclonal antibodies that inhibit LDLR degradation and reduce LDL [174], consistent with the clustered regularly spaced short palindromic repeats (CRISPR) and CRISPR-associated protein (Cas) system-mediated silencing of PCSK9 in non-human primates, where LDL was reduced by 60% [175].

### 3.2. IDOL Regulation of LDLR and LDL

IDOL is an E3 ubiquitin ligase involved in LDLR degradation. Untreated FH patients had higher serum levels of IDOL and PCSK9 than the control, and serum IDOL levels decreased after statin therapy, suggesting statins’ role in decreasing serum IDOL [176]. This was further confirmed in an in vitro assay which showed that atorvastatin significantly decreased IDOL abundance in a dose-dependent manner in cultured macrophages and hepatocytes with a concomitant increase in LDLR expression [176]. Making LDLR resistant to PCSK9 and IDOL-mediated degradation by introducing mutation in the LDL receptor conferred partial resistance to LDLR degradation and reduced LDL cholesterol levels in mice [177]. These studies further demonstrate that LDLR protein levels are associated with circulating LDL. Similar to statins, bempedoic acid, an ATP citrate lyase inhibitor, also works by inhibiting cholesterol synthesis in cell-based assays [8] and animal models [9]. In human hepatocytes, bempedoic acid showed inhibition of cholesterol synthesis as well as increases in LDLR activity [61]. Another protein, inducible degrader of LDL receptor (IDOL), also increases LDL receptor degradation by a different mechanism [178] which was shown to be regulated by LXR. IDOL knockdown in hepatocytes increased LDLR protein, and IDOL overexpression enhanced LDLR degradation [179]. Statins also suppress IDOL in human hepatoma cells, leading to LDLR upregulation [18]. In vivo depletion of IDOL in IDOL knockout hamsters showed protection against atherosclerosis in hamsters [180]. On the contrary, overexpression of IDOL in mice and hamsters reduced liver-specific LDLR expression and elevated apoB-containing atherogenic lipoproteins [181]. Other pathways have also been described that target the liver for the treatment of hypercholesterolemia [182].

## 4. Management of Hypercholesterolemia via ApoC-III and ANGPTL3 Inactivation

Loss-of-function phenotypes of both apoC-III [116,117,119] and ANGPTL3 [22,55,127] genes in humans share common features in terms of their mode of action on TG-rich apoB lipoproteins (TRLs). Both are expressed in the liver and play an important role in the metabolism of apoB lipoproteins by facilitating hepatic secretion of TG-rich lipoproteins and impairing the clearance of highly atherogenic TRLs via inhibition of lipoprotein lipase [183] (Figure 3). ApoC-III loss-of-function mutation is associated with low risk of ischemic heart disease and coronary disease [119,184,185]. Targeting of apoC-III to inactivate its activity by volanesorsen, an siRNA, showed significant reductions in plasma TG levels in hypertriglyceridemic [57,58] and partial lipodystrophy [20] patients. Severe hypertriglyceridemic patients showed remarkable reductions in TG levels following treatments with apoC-III ASO [57,58]; however, in some studies, thrombocytopenia side effects are reported [120]. A novel siRNA, ARO-APOCIII, targeting apoC-III is currently in a late-stage clinical evaluation for lipid-lowering efficacy and overall safety profile with respect to thrombocytopenia [120]. In both diabetic [113] and non-diabetic [114] patients, treatment with apoC-III ASO, volanesorsen, significantly reduced plasma apoC-III and TG, but unlike ANGPTL3 inhibition, it raised HDL compared with a placebo. In diabetic patients, these changes in apoC-III and TG were accompanied by a 57% improvement in whole-body insulin sensitivity (*p* < 0.001). Importantly, a strong correlation between enhanced insulin sensitivity and both plasma apoC-III and TG suppression was observed together with lower HbA1c. An increased apoC-III/HDL ratio was shown to be associated with CAD as a result of impaired cholesterol efflux capacity [186], suggesting that reducing the apoC-III/HDL ratio through targeting apoC-III by ASO may prove to be protective against CAD. Given the higher TG and apoC-III in diabetic patients, it is expected that the cholesterol efflux capacity in diabetics would be lower compared to age-matched non-diabetic individuals. Indeed, multiple studies suggest that cholesterol efflux capability in diabetes is impaired [69,187]. 

ANGPTL3 is also involved in the hepatic secretion of TG-rich lipoproteins and clearance of TRLs via inhibition of LPL [60]. Loss-of-function mutations in the ANGPTL3 gene in humans have shown an association with lower TG and LDL levels and reduced risk of ASCVD [22]. Antisense oligos [21] as well as monoclonal antibodies [22] have been developed to inactivate ANGPTL3 in humans. Comprehensive results have been published on genetics and mAb-mediated inactivation of ANGPTL3 in multiple animal species as well as in humans [22], and the findings have been generally consistent across species in terms of lipid lowering and ASCVD risk reduction. Treatment of FH patients carrying loss-of-function LDLR mutations (HoFH, HeFH, compound FH) with ANGPTL3 mAb for 4 weeks showed remarkable mean reductions of 50% (27 to 61) in LDL and 47% in TG [52]. In a double-blind placebo-controlled dose-ranging phase 2 study, vupanorsen, an siRNA targeted to liver ANGPTL3, was evaluated in 105 patients with symptoms of triglyceridemia, diabetes and steatohepatitis [21]. While the patient profile in this study in terms of higher levels of TG-rich lipoproteins is certainly a good idea, inclusion of steatohepatitis and diabetes may not be something that is expected to be improved by inhibiting the activity of an agent such as ANGPTL3 that would inhibit egress of lipids from the liver. In this scenario, although plasma TG showed a 44% reduction in the highest dose group (80 mg) tested, LDL cholesterol decreased by only 7%. No significant changes were seen in any of the other parameters associated with diabetes, such as HOMA, fatty acids and HbA1c. The hepatic fat fraction in the highest dose group increased significantly, suggesting that lipids are retained in the liver, as observed in FHBL in individuals that causes fatty liver [101]. Moreover, these FHBL individuals are at risk of developing diabetes [104]. The clinical data are consistent with the mechanism of action of ANGPTL3 in hepatic lipid secretion. Wang et al. [126] have shown that inactivation of ANGPTL3 inhibits secretion of VLDL-TG, but not VLDL-apoB. In addition, the hepatic TG in these mice remained unchanged in a fed state. Fat accumulates in the liver when its egress into circulation is impaired by inactivation of ANGPTL3. However, studies of ANGPTL3 inhibition show that it also lowers HDL [21,22,188] via activation of endothelial lipase. Loss-of-function mutations of ANGPTL3 in humans also show low levels of HDL compared to non-carriers [127]. It is possible that low levels of HDL may impact reverse cholesterol transport (RCT). To assess HDL functionality, a systematic dose-response study was carried out to inactivate ANGPTL3 using antisense oligos in mice [189]. In animal models (WT, LDLR^−/−^, CETP-Tg/LDLR^−/−^), treatment with ANGPTL3 antisense oligo caused HDL lowering but increased RCT, as evidenced by >two-fold increases in fecal cholesterol. Thus, ANGPTL3 inactivation increased RCT despite lowering of HDL and showed no correlation between HDL level and RCT, similar to other findings [190], demonstrating the importance of HDL function over HDL concentration and the possible involvement of other players influencing the elimination of body cholesterol into feces [191]. 

## 5. LDLR Gain-of-Function Mutations

**Figure 4:**
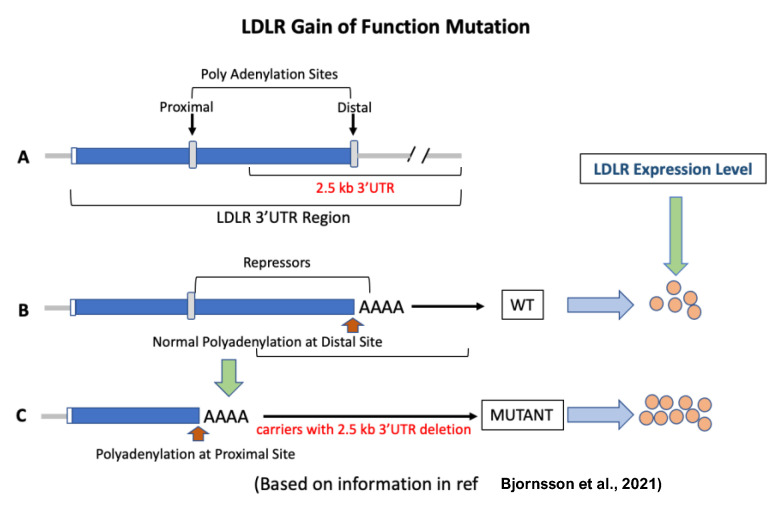
LDLR gain-of-function mutation [194]. (**A**): The LDLR gene has two polyadenylation sites. The 2.5 kb DNA segment flanks the distal site. (**B**): Normally, polyadenylation occurs at the distal site, and as a result, the binding sites for repressors of the LDLR gene remain intact. (**C**): When the 2.5 kb 3′UTR region is missing, polyadenylation occurs at the proximal site. This results in the upregulation of LDLR gene expression by 2-fold and a circulating LDL reduction of 74% in carriers compared to non-carriers of the mutation.

Studies from LDLR knockout (loss-of-function) [13] and LDLR transgenic (gain-of-function) [135] mice have clearly established the association of LDLR activity and circulating LDL levels. Similar findings were reported in hetero- and homozygous hamsters [155] and pigs [165]. While LDLR loss-of-function mutations have been widely reported for the past 5 decades, an LDLR gain-of-function study was reported only recently in an Icelandic population. LDL receptor defect or dysfunction leads to an increased level of LDL and premature cardiovascular disease, as demonstrated by high impact loss-of-function mutations in the LDLR gene that cause familial hypercholesterolemia [192]. In contrast, gain of LDLR function would be expected to lower circulating LDL levels (Figure 2). A splice region variant of the LDL receptor has been identified [193] that results in lowering of non-HDL cholesterol (apoB-containing atherogenic lipoproteins) and protection from development of coronary artery disease. This splice variant is located within intron 1 with a strong enhancer activity in HepG2 cells and shows higher LDLR mRNA expression in white blood cells isolated from the affected individuals. The other gain of function of LDLR in the Icelandic population was reported in 7 heterozygous carriers from a single family, which occurred as a result of deletion of the 2.5 kb 3′ untranslated region of the LDLR gene [194] (Figure 4). The deletion of this 2.5 kb 3′UTR of LDLR abolishes the binding sites of miRNAs that negatively influence the gene expression [89,90,91,92,93]. The removal of the 3′UTR region of the LDLR gene that harbors binding sites of repressors resulted in an elevation of LDLR activity by 1.8-fold and mean LDL cholesterol lowering by 74% compared to non-carriers (Figure 4). These individuals are protected from developing cardiovascular disease. Thus, the gain-of-function mutation of LDLR in the human population provides further proof of concept that LDLR activity is tightly associated with circulating LDL and is causative in terms of providing protection from developing coronary artery disease.

## 6. Gene Therapy and Genome/Base Editing Approaches to Manage Refractory Hypercholesterolemia

Patients with refractory hypercholesterolemia (RH), often seen in hereditary metabolic disorders like HoFH and compound heterozygous familial hypercholesterolemia (HeFH), have significantly elevated LDL levels despite treatment with maximum tolerated doses of lipid-lowering therapies. These patients are at a very high risk for developing ASCVD [195,196]. HoFH, primarily a monogenic autosomal-dominant disorder, causes marked elevation of plasma LDL and increases the risk of developing severe CVD during the early life-span (<20 years of age), which requires invasive therapies and may ultimately lead to a heart transplant. LDLR-dependent therapy with statins results only in modest reductions in LDL (10–25%) [197], and combination therapy with ezetimibe, an LDL-independent therapy, lowers LDL by up to 30% [197]. Given the remarkably high level of LDL (>400 mg/dL) in HoFH patients, the 30% reduction in LDL is not enough to reduce the LDL to a level sufficient to provide protection from CVD risk. Recent LDLR-dependent therapies such as PCSK9 mAb are not effective in HoFH but are able to reduce LDL by 25% in HeFH with at least one functional LDLR allele [198]. Other LDL-lowering therapies are associated with side effects. ANGPTL3 mAB therapy did reduce LDL in HoFH by an impressive 50% [52] but failed to achieve its therapeutic goal. The highest reduction of 80% in circulating LDL was reported via liver transplant [199], which does not appear to be a practical solution. These obstacles in achieving therapeutic LDL goals require HoFH patients to get weekly or biweekly lipid apheresis performed on a regular basis [200]. Since high LDL levels in most of the HoFH patients are caused by a single gene loss-of-function mutation, mitigating the defective gene by introducing a functional copy of the gene could provide a permanent solution to maintaining therapeutic levels of LDL. This concept has been tested in LDLR-deficient mice using recombinant viral vectors such as a retrovirus [201], lentivirus [202,203], helper-dependent adenovirus [204] and adeno-associated virus [205]. Although the mouse lipoprotein profile differs from that of humans—for instance, the lack of CETP and HDL being a major circulating lipoprotein—there appears to be a marked similarity between mice and humans with regard to LDLR-mediated clearance of LDL lipoproteins. Loss-of-LDLR-function mutation raises LDL in both mice and humans [1,2,3,13] and overexpression of LDLR reduces LDL in mice [135,206]. It should be noted that LDLR-deficient mice (HoFH and HeFH) require feeding an HFHC diet to raise LDL to a level seen in humans to cause atherosclerotic lesions [13]. Transplantation of autologous ex vivo genetically modified hepatocytes using recombinant retroviruses carrying healthy LDLR infused into patients’ livers directly did not achieve the expected level of efficacy and appears to be impractical and associated with risks. An adeno-associated virus (AAV), especially the AAV8 serotype, has been found to be better in terms of transduction efficiency as well as humoral immunity and T cell response to the capsid [207]. Recent advancements in gene therapy for the treatment of hypercholesterolemia focused on the codon optimization of the AAV vector have shown encouraging results in LDLR^−/−^/apobec1^−/−^ double knockout mice [207,208] (Table 2). In order to overcome host immune response to viral vectors and lower toxicity and immunogenicity, minicircle non-viral DNA vectors were created that resulted in highly efficient liver-specific LDLR gene expression in LDLR-deficient mice and produced marked reductions in LDL with no significant toxicity [209]. 

Recent approaches utilizing CRISPR-Cas9 technology have opened up opportunities to correct causative loss-of-function mutations both ex vivo and in vivo [210,217]. Ex vivo correction of the gene defect using viral vectors and reintroduction into patients has its own challenges [201]. The CRISPR-Cas9 approach involves designing artificial single-guide RNA (sgRNA) to orient and to recognize the sequence at the targeted location in the genome, followed by the cleavage of the DNA double strand by Cas9 nuclease. Subsequently, the cell’s natural nonhomologous end-joining repair machinery joins and restores the original DNA sequence or introduces insertion or deletion (indel mutation). Both AAV [218] and non-viral [219] vehicles have been used for the inactivation of PCSK9 by the CRISPR-Cas9 system in mice or in human cells lacking the LDLR gene [220,221], with encouraging results (Table 2). The direct CRISPR/Cas9 in vivo approach is potentially feasible, since it has been tested in animal models with great success [210]. The results of evidence-based research strongly suggest that the CRISPR-Cas9 system is becoming a strong tool for gene engineering to create genetically modified animal models [155,175,210] for therapeutic agent testing and extending of this technology to humans to treat diseases arising from genetic defects [222]. 

A gene-editing drug product contains recombinant enzymes and guide RNAs for the gene editing, formulated in either lipid nano particles (LNP) [212,223] or packaged in AAVs [224], with a primary goal to lower LDL in hypercholesterolemic patients. This technology has been shown to work in animal models for a number of genetic diseases [222]. CRISPR-Cas9 technology to correct the LDLR gene mutation causing LDLR loss of function has been successfully evaluated in hamsters [155], primates [175] and mice [210]. Zhao et al. [210] first generated an LDLR loss-of-function mouse model by introducing a nonsense point mutation using CRISPR base editing technology that caused a remarkable increase in LDL cholesterol on a high-fat diet and severe atherosclerosis in the aortae. Treatment of these hypercholesterolemic mice with CRISPR/Cas9 editing components packaged in an AAV8 vector showed high-efficiency transduction of hepatocytes that resulted in partially restoring the LDLR function and lowering LDL by >60% (Table 2). The extent of the reduction in aortic atherosclerosis in the treatment group paralleled reductions in LDL [210], confirming the proof of concept using the gene-editing CRISPR/Cas9 system to correct a disease-causing mutation. While the CRISPR-Cas9 system offers opportunities to treat hypercholesterolemia and other rare genetic diseases [222], the major concerns are the unpredictable random insertion or deletion at the target site and off-target mutagenesis because of lack of sufficient specificity of guide RNA. These concerns may limit the application of genome editing for clinical purposes. FDA guidelines recommend that all biotechnology/gene-therapy-derived products be evaluated for proof of concept, efficacy and safety of the drug product in appropriate animal models before testing them in humans. The most important criteria for pharmacology and safety evaluation are (a) the selection of appropriate animal models, where the product retains its biological activity; (b) the pharmacokinetic property and biodistribution of the drug product; (c) relevance of the animal models in humans when it comes to the dose–response relationship and monitoring of pharmacology and safety biomarkers; and (d) off-target gene modification and related safety concerns. A number of studies relating to antisense, mAb and gene therapy/gene editing drug products utilize non-human primates for efficacy and safety studies [95,169,175,225]. 

**Figure 5:**
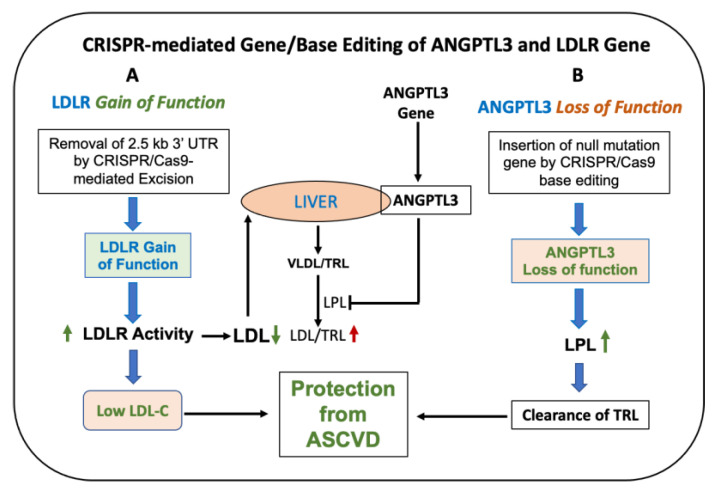
CRISPR-mediated gene/base editing to lower LDL in hypercholesterolemic patients. (**A**): CRISPR/Cas9-mediated excision of the 2.5 kb 3′UTR region of LDLR in FH patient-derived cells and in mice increases LDLR activity and mRNA [211]. (**B**): Base editing of ANGPTL3 resulting in the loss of function decreases LDL by enhanced clearance as a result of LPL activation. Inactivation of ANGPTL3-mediated lowering of LDL occurs via an LDLR-independent pathway. Green shows protection from CVD. Red arrow indicates increase in CVD risk.

The new generation of CRISPR-Cas9-mediated gene editing is called base editing, where undesired off-target gene modifications can be avoided by utilizing the positioning and cleavage capability of the Cas nuclease to yield precise single-base substitution without the need for DNA cleavage [226]. Two classes of base editors, cytosine base-editors (CBE, C → T, T → C) and adenine base editors (ABE, A → G, G → A), have been described [227]. Less off-target mutagenesis was noticed with the 3rd generation base editor (BE3) when compared to the CRISPR-Cas9 system [228]. In vivo base editing to inactivate the PCSK9 gene in mice showed an indel mutagenesis rate of only ~1% [213] compared to ~40% mutagenesis reported in in vivo genome editing [229] (Table 2). A follow-up study to inactivate the ANGPTL3 gene was carried out using an AAV vector encoding BE3 and gRNA to target codon Gln-135 in C57Bl/6J and LDLR^−/−^ mice [214]. More than 50% reductions in cholesterol and TG were reported in LDLR^−/−^ mice, with no off-target mutagenesis. The precision of base editing as well as lack of off-target mutagenesis were also demonstrated in PCSK9 humanized mice [230]. Liver-specific base editing of the PCSK9 gene in primates using lipid nanoparticles (LNP) resulted in 90% and 60% reductions in blood PCSK9 and LDL for up to 8 months, respectively [175], showing a clear association between LDLR and plasma LDL level and the validity of CRISPR base-editing technology to precisely edit a codon with very high efficiency and without off-target safety issues. Another study also demonstrated base editing by ABEmax both in mice and macaques [215]. In a similar CRISPR base-editing system, the ANGPTL3 gene was successfully inactivated in non-human primates when administered as lipid nanoparticles [216,225]. In this study, a single 3.0 mg/kg dose maintained 54–57% editing and suppression of blood ANGPTL3 levels by >95% for up to 2 years, with no sign of off-target safety issues in the liver. In a separate study with LDLR-deficient NHP, 64% editing was achieved that resulted in an 87% reduction in blood ANGPTL3 levels [216] (Figure 5). 

These results using the CRISPR base-editing technology established proof of concept in NHP [89,90,91,92,93] and offer hope to hypercholesterolemic patients. Another approach to enhance LDLR activity was proposed from the results of a human genetic study recently reported in an Icelandic population that showed elevated LDLR activity in carriers missing the 2.5 kb 3′UTR region of the LDLR gene [194]. The missing sequences contain binding sites of miRNAs [89,90,91,92,93] known to repress LDLR expression. Using CRISPR-based genome editing technology and a proprietary nuclease, a group of researchers was able to successfully delete the 2.5 kb 3′UTR region of the LDLR gene in an FH patient-derived lymphoblastoid cell line and in mice that resulted in increased LDLR mRNA, protein and activity [211] (Figure 5), similar to that observed in human carriers lacking this same 2.5 kb 3′UTR region of the LDLR gene [194]. This is the first example of directly targeting the LDLR gene in vivo to enhance LDLR activity based on natural mutations. The proof of concept in humans offer hope to hypercholesterolemic patients. This approach appears to eliminate any liver safety issues associated with other therapies that inhibit egress of lipids from the liver. However, safety issues relating to off-target mutagenesis of this approach need to be demonstrated. Such a demonstration should provide a concise and precise description of the experimental results, their interpretation, as well as the experimental conclusions that can be drawn. 

## 7. Conclusions

Ample literature on LDLR activity and circulating LDL levels in animal models and clinical studies, together with in vitro studies, shows the association between LDLR activity and circulating LDL. In the majority of clinical situations where patients were treated with LDLR-raising therapy and also in animal studies where the LDLR gene was ablated (heterozygous or homozygous) and treated with LDLR-modifying agents, a tight association between LDLR activity and LDL was observed. However, in certain pathophysiological conditions such as diabetes and oxidative stress, other factors may weaken LDL and ASCVD risk correlations. Preclinical and clinical results demonstrate that gain of function by gene therapy and genome/base editing of LDLR-based therapy may be better in terms of management of hypercholesterolemia and overall safety pharmacology. While targeting lipid synthesis in the liver has shown marked benefit in lowering blood LDL, the inhibition of hepatic lipid secretion, although it improves circulating LDL and TRL, is often associated with accumulation of lipids in the liver. LDL clearance pathways via directly targeting LDLR or via other players, such as PCSK9, have shown benefits to HoFH and HeFH patients, respectively. Two approaches utilizing CRISPR-based gene/base editing currently underway to enhance clearance of LDL and TRL by targeting LDLR and ANGPTL3, respectively, offer great promise to help hypercholesterolemic patients achieve the therapeutic goals of LDL. One caveat of the enhancing LDLR expression approach is that one needs at least one functional LDL allele to target. Thus, only HeFH patients, and possibly other hypercholesterolemic patients with an intact LDLR gene, are likely to benefit. Based on non-human primate results, ANGPTL3 inactivation using CRISPR base editing appears promising to HoFH patients, once the safety of this approach is demonstrated in clinical studies. 

## Figures and Tables

**Table 2:** Preclinical studies aiming at validation and optimization of new approaches targeting LDLR-dependent and -independent pathways to lower LDL.

Preclinical Studies	Drug Class	Primary Endpoint	Mechanism	Refs.
Adenoviral	Gene therapy	LDL	LDLR expression	[205,207]
AAV	Gene therapy	LDL	LDLR expression	[208]
AAV/CRISPR-Cas9	Gene editing	LDL	LDLR expression	[210]
CRISPR-Cas9	Gene editing	LDL	LDLR expression	[211]
LNP/CRISPR-Cas9	Gene editing	LDL, TG	ANGPTL silencing	[212]
CRISPR-Cas9	Base editing	LDL	PCSK9 silencing	[213]
CRISPR-Cas9	Base editing	LDL, TG	ANGPTL silencing	[214]
CRISPR-Cas9	Base editing	LDL	PCSK9 silencing	[215]
CRISPR-Cas9	Base editing	LDL, TG	ANGPTL silencing	[216]

## Data Availability

No new data reported.

## Abbreviations

AAV	adeno-associated virus
ABE	adenosine base editors
ACLY	ATP citrate lyase
ANGPTL3	angiopoietin-like 3
Apo	apolipoprotein
ASO	antisense oligo
ASCVD	atherosclerotic cardiovascular disease
CAD	coronary artery disease
CBE	cytosine base editors
CHD	coronary heart disease
CETP	cholesteryl ester transfer protein
CRISPR	clustered regularly interspaced short palindromic repeats
FHBL	familial hypobetalipoproteinemia
HCHF	high cholesterol high fat
HeFH	heterozygous familial hypercholesterolemia
HMG-CoA	β-Hydroxy β-methylglutaryl-CoA
HoFH	homozygous familial hypercholesterolemia
HOMA	homeostatic model assessment
IDOL	inducible degrader of LDLR
KO	knockout
LDLR	low-density lipoprotein receptor
LCAT	lecithin:cholesterol acyltransferase
LNP	lipid nanoparticles
Lp(a)	lipoprotein(a)
LPL	lipoprotein lipase
mAb	monoclonal antibody
NPC1L1	Niemann–Pick C1-Like1
oxLDL	oxidized LDL
PCSK9	proprotein convertase subtilisin/kexin type 9
RH	refractory hypercholesterolemia
TRL	triglyceride-rich lipoproteins
UTR	Untranslated region
WHHL	Watanabe heritable hyperlipidemic
WT	wild type
